# Comparative Efficacy and Safety of Two Different Formulations of Linear Hyaluronic Acid in Patients with Knee Osteoarthritis

**DOI:** 10.3390/ph18071065

**Published:** 2025-07-19

**Authors:** Vincenzo Rania, Cristina Vocca, Gianmarco Marcianò, Maria Cristina Caroleo, Lucia Muraca, Emanuele Toraldo, Francesca Greco, Caterina Palleria, Gian Pietro Emerenziani, Luca Gallelli

**Affiliations:** 1 Operative Unit of Pharmacology and Pharmacovigilance, “Renato Dulbecco” University Hospital, 88100 Catanzaro, Italy; raniavincenzo1@gmail.com (V.R.); palleria@unicz.it (C.P.); 2 Department of Health Science, University Magna Graecia of Catanzaro, 88100 Catanzaro, Italy; cristina_vocca@live.it (C.V.); gianmarco.marciano3@gmail.com (G.M.); mariacristina.caroleo@unicz.it (M.C.C.); 3 Research Center FAS@UMG, Department of Health Science, University Magna Graecia of Catanzaro, 88100 Catanzaro, Italy; lalumuraca@gmail.com; 4 Department of Clinical and Experimental Medicine, Catanzaro University Magna Graecia of Catanzaro, 88100 Catanzaro, Italy; emanuele.toraldo@studenti.unicz.it (E.T.); f.greco@unicz.it (F.G.); emerenziani@unicz.it (G.P.E.); 5 Medifarmagen, “Renato Dulbecco” University Hospital, 88100 Catanzaro, Italy

**Keywords:** hyaluronic acid, triple injection, linear, knee osteoarthritis, safety, efficacy

## Abstract

**Introduction**: Knee osteoarthritis (OA) is defined by articular cartilage loss, increased discomfort, and functional restrictions. Changes in lifestyle, painkillers, intra-articular injections, and, as a last resort, surgery are all part of clinical therapy. In this setting, intra-articular injections of hyaluronic acid (HA) represent a relevant and diffused therapeutic option. **Materials and Methods**: A prospective observational study was performed from October 2024 to May 2025 in 70 patients with knee OA. HA was administered in three intra-articular injections and was followed up at 3 and 6 months from the last injection. Knee Injury and Osteoarthritis Outcome Score (KOOS) was evaluated as primary outcome measure; Visual Analogue Scale (VAS), time up and go test, six-minute walking test, general health assessment with 36-Item Short Form Health Survey (SF-36), Zung’s Self-Rating Anxiety Scale (Zung SAS), and Zung’s Self-Rating Depression Scale (Zung SDS) as secondary outcome measures. **Results**: We observed a statistically significant improvement in clinical scores at 3 months in both HA formulations compared to the control group. No relevant side effects were described during the study. **Conclusion**: Hyalubrix 30 mg/2 mL and DIART 1.8%/2 mL are two safe and effective therapeutic options to manage knee OA, offering benefits in pain control, functionality and emotional wellness.

## 1. Introduction

Knee Osteoarthritis (OA) is a clinical condition characterized by progressive cartilage degeneration in the joint, with narrowing of the joint space and increased direct bone contact [1].

This pathology is very common in the general population. In 2020, the prevalence of knee OA in people of 15 years or older was 16.0% and its incidence was 203 per 10,000 person-years worldwide. Incidence and severity usually increase with age and OA is more common in females than males [2]. Several factors may favor the emergence and progression of OA including obesity, sports, traumatic injury, and some types of work (e.g., construction) [3,4,5,6].

Clinical symptoms are generally intermittent pain and stiffness, potentially associated with instability and swelling [7,8]. Knee OA is diagnosed through clinical examination and radiography. Further information may be obtained using other imaging studies including ultrasound, magnetic resonance imaging, and computed tomography, although these are not mandatory [9].

The progression of OA gradually causes bone osteophyte formation and sclerosis of the subchondral bone, and in advanced cases, subchondral cyst formation [10]. The main therapeutic option for severe knee OA is total joint replacement (TJR). Nevertheless, performing surgery is not always possible (especially in the elderly) and not all patients are willing to undergo it. The pharmaceutical industry is focusing on new therapeutic options to repair cartilage, restoring its function and reducing pain [11].

The management of knee OA depends on the stage and on patients’ symptoms, using both pharmacologic and non-pharmacologic approaches. The prescription of analgesic drugs (e.g., non-steroidal anti-inflammatory drugs, NSAIDs) is very common, but it is associated with the development of side effects in cases of abuse. Therefore, the prescription of intra-articular injections is very useful to reduce the number of systemic side effects and to achieve clinical efficacy acting directly on the target [12,13,14,15,16,17]. The most important therapeutic options in this setting are corticosteroids, platelet-rich plasma (PRP) and hyaluronic acid (HA) [12,18].

HA acts in a rheological manner in synovial fluid and cartilage. It results in protection of cartilage, lubrication, anti-inflammatory effect and shock absorption of the joint [19]. Different formulations of HA are available on the market. They differ in molecular weight (low or high), number of injections, production source and cross-linking [20,21,22]. The main aim of HA injections is not only symptom improvement, but also delaying TJR [23] in a dose-dependent fashion, by improving joint lubrication, reducing inflammation, and providing pain relief [24].

In fact, since the effects of HA on the delay of TJR are controversial, a recent study reported that hyaluronic acid injections are generally safe, even if local pain and swelling are the main side effects [19,25,26].

There is currently no conclusive evidence on which of the two formulations of HA—one injection or three—is more helpful in alleviating symptoms. In practice, a single injection lessens adverse effects and patient discomfort, whereas three injections provide a better chronological distribution and a more focused patient follow-up [19].

In this clinical study, we compared the efficacy and safety of Hyalubrix 30 mg/2 mL and DIART 1.8%/2 mL, two different formulations of linear hyaluronic acid, administered in a three-injection regimen.

## 2. Results

### 2.1. Patients

Seventy knee OA patients were enrolled in the trial and randomly assigned to receive either DIART 1.8%/2 mL or Hyalubrix 30 mg/2 mL (Table 1). The majority of patients in this study had grade III Kellgren–Lawrence knee OA (62.9%) and were women (54.3%). The most prevalent comorbidities were hypertension (41.4%) and osteoporosis (35.7%). The DIART 1.8%/2 mL group had significantly greater rates of dyslipidemia, hypertension, and diabetes. MANOVA tests excluded a role of comorbidity in HA effects. Enrolled individuals consumed analgesics before starting treatment, mostly NSAIDs in the Hyalubrix 30 mg/2 mL group (Table 2).

### 2.2. Control Group

Fifty patients with OA and with similar characteristics as the test groups were assigned to the control group and received intra-articular triamcinolone acetonide (40 mg/mL) and lidocaine (20 mg/mL; 2 mL) (three intra-articular injections every ten days; one injection every week for three weeks) and were monitored for 6 months (Table 3).

### 2.3. Effects on Pain

At T0 the mean values of VAS (control group 6.78 ± 1.35; Hyalubrix 30 mg/2 mL: 7.69 ± 0.9; Diart 1.8%/2 mL 7.89 ± 0.9) and KOOS (control group 46.35 ± 18.36; Hyalubrix 30 mg/2 mL: 47.34 ± 14.31; Diart 1.8%/2 mL 49.49 ± 15.68) were similar between the groups. In both groups, the walking test was positive after 3 min, suggesting the presence of cartilage damage. Timed up and go test at this time was 5.4 ± 2.3 s in the control group, while 9.37 ± 2.18 and 9.17 ± 2.42 in the Hyalubrix 30 mg/2 mL and Diart 1.8%/2 mL groups, respectively. In all groups, we documented a significant time-dependent (T0-T3) improvement in VAS, KOOS, and timed up and go test in HA groups (Table 4). According to statistical analysis, the administration of Hyalubrix 30 mg/2 mL did not result in any improvement in clinical pain at T5 when compared to Diart 1.8% 2 mL (Table 5). In the control group, we documented significant impairment at T3 (Table 6).

### 2.4. Effect on Quality of Life and Mood Symptoms

Using SF-36 (quality of life), Zung SDS (depression) and Zung SAS (anxiety) scales, we observed a statistically significant improvement in quality of life and mood disorders (*p* < 0.01) in both groups after the treatment and during the follow-ups. Moreover, statistical analysis of SF-36 and Zung’s Depression and Anxiety Scales at 3 weeks (T3) and 3 months (T4) showed that both HAs were effective at improving patients’ outcomes (Table 7 and Table 8).

At 6 months (T5) secondary outcomes were all still significant, except social functioning for Hyalubrix 30 mg/2 mL and role limitations due to physical health and Zung’s depression scale for DIART 1.8%/2 mL (Table 9). In the control group, drug treatment (triamcinolone acetonide plus lidocaine) improved both quality of life and mood symptoms after the first administration (T1), but we recorded impairment during the follow-ups (T3) (Table 10). In both the HA treatment groups, the MANOVA test did not show a statistically significant association between comorbidity and pain; it only revealed a statistically significant correlation between age and pain.

### 2.5. Safety

During this study, we did not record any adverse drug reactions. Furthermore, as of June 2025, we have not documented any adverse drug responses that occurred in enrolled patients subsequent to the study.

## 3. Discussion

In this study we compared the efficacy and safety of two linear formulations of hyaluronic acid (molecular weight > 1500 kDa), Hyalubrix 30 mg/2 mL and DIART 1.8%/2 mL, documenting that both formulations improved patients’ clinical conditions without clinically relevant ADRs. The use of topical treatments plays an important role in the management of knee OA, as it helps reduce the number of oral drugs used and allows for targeted action at the joint level [27]. All enrolled patients previously consumed analgesic drugs without clinical effect and received triamcinolone injection to reduce inflammation and facilitate the action of HA, but without significant clinical benefit. The reduction of drugs like NSAIDs and corticosteroids is very helpful in reduce the relevant side effects like nephrotoxicity, cardiotoxicity with NSAIDs and hypertension, diabetes and osteoporosis with corticosteroids [17,28,29].

It is noteworthy that both formulations have similar pharmacological activity, showing comparable pharmacokinetics with a similar half-life after intra-articular administration, as well as similar pharmacodynamics. However, the DIART 1.8%/2 mL formulation, containing HA combined with succinate, may provide additional benefits related to the anti-inflammatory and antioxidant properties of succinate, alongside its potential role in enhancing cellular energy metabolism through stimulation of the Krebs’ cycle. Regarding the mechanism of action, HA exerts its effect not only through rheological properties but also by mediating anti-inflammatory action (e.g., suppressing pro-inflammatory cytokines and chemokines, promoting the synthesis of anti-inflammatory mediators) [30,31,32,33,34].

Moreover, the interaction of HA with its main receptor, CD44, causes several beneficial effects: anti-inflammatory, subchondral, chondroprotective, and proteoglycan production effects, with a decrease in cytokines/metalloproteinases and a reduction in joint cartilage disruption [35].

Our results reflect this action, showing the complete efficacy of both formulations.

It is important to consider some differences between the groups: (i) in both groups females were more numerous than males; (ii) female sex is generally associated with a higher severity of knee OA; (iii) most of our patients were middle-aged. This latter point is relevant because the severity of OA tends to increase in elderly people, particularly in women after menopause [36,37,38].

In an interesting review, Wakale et al. [39] reported that in elderly patients, the cumulative effect of risk factors like senescence, altered epigenetics, mitochondrial dysfunction, changes in cell metabolism, and growth factor response affects chondrocyte homeostasis and is involved in the development of OA. Moreover, cartilage is an estrogen-sensitive tissue, showing sex differences in cartilage degeneration and repair [40,41].

During menopause, women experience a decline in estrogen levels [38]. The significant rise in OA prevalence among postmenopausal women suggests a correlation between OA and loss of estrogen [42].

Furthermore, in the DIART 1.8%/2 mL group, there were patients that had both knees affected by OA and were both managed. This fact may have accounted for higher clinical severity and worsening clinical and, in particular, functional outcomes. On the other hand, the presence of a higher number of subjects with osteoporosis, fibromyalgia and anxiety/depression may have caused worse results for Hyalubrix 30 mg/2 mL, especially considering Zung’s scales [43,44,45,46]. Diabetes was more frequent in patients who were managed with DIART 1.8%/2 mL (*p* < 0.05), and in case of diabetic neuropathy, the referred NRS may be affected by this clinical condition [47]. In both groups, most subjects were affected by grade III knee OA.

Hyalubrix 30 mg/2 mL efficacy was previously widely documented in literature, even in other joints like hip and shoulder [48,49]. The effects on the knee are largely proven. Foti and colleagues [50] recruited 1266 patients with OA (including knee OA) in 47 centers. The knee was the most affected joint in this population and all patients received Hyalubrix 30 mg/2 mL, whose efficacy was evaluated through VAS, Health Assessment Questionnaire (HAQ) and the Euro Quality of Life (QoL) questionnaire. Statistically significant improvements were observed (*p* < 0.0001). In this cohort, 0.8% of patients reported adverse events for a total of thirteen events (mainly of mild/moderate intensity). Similar results were observed by Giarratana et al. [51] with 72 patients. In this group, pain and KOOS improved in a statistically significant manner from T0 to 26 weeks.

In our study, Hyalubrix 30 mg/2 mL was completely effective in managing pain, but lost its efficacy by 6 months compared to DIART 1.8%/2 mL. This is probably mainly due to time-related degradation of HA.

Quality of life significantly improved in both groups compared to baseline, with few differences concerning social functioning, depression symptoms and role limitations due to physical health. This is one of the few papers analyzing the modifications in patients’ mood after HA supplementation. Considering the presence in the two groups of a few subjects with depression and anxiety, the results are good. The relationship between pain, mood disorders and impairment of quality of life has been widely described in the literature. Acting on pain is useful to improve these features [26,52].

The presence of different HA formulations raises the question of evaluating the most effective formulation considering cross-linking and number of injections. A recent paper by Safali et al. [19] compared two different dosages of HMW HA (SEMICAL^®^) triple 30 mg injections with a one-week interval and 60 mg single injections in 128 patients. Lequesne Score, VAS and Western Ontario and McMaster Universities Osteoarthritis (WOMAC) Index were evaluated, showing more favourable results for the 30 mg formulation (follow-up period of a year), despite both formulations being highly effective. Nevertheless, this result may be associated with this specific formulation only and the different total dosages (90 mg vs. 60 mg). Other reasons indicated by the authors are the sustained released of HA over multiple doses with repeated anti-inflammatory and chondroprotective effects, and better HA distribution related to low dose injections. Nevertheless, the authors themselves recognize the multiple limitations of their study including the retrospective design, absence of a comparison group with a different molecule, and recruitment of a population in the 50–60 years range. This paper is in disagreement with other previous studies, like that of Conrozier et al. [53] showing comparable efficacy of single vs. triple injection of HMW HA (hylan G-F 20) with the same total dosage. The advantage of a single injection becomes clear in patients managed with anticoagulants, busy subjects or those not tolerating three injections. A minority of authors, including Petterson and colleagues, observed superiority of single injection HA [54]. In our experience, single injection may be useful to increase patients’ compliance and reduce side effects [26]. Probably, three injections offer better coverage and are generally considered the best choice. Nevertheless, there are many different formulations of HA and drawing definitive conclusions is difficult without directly comparing two single formulations in high numbers of patients.

Our study has some limitations. Firstly, the number of patients was relatively low. Secondly, we collected data only for 6 months, whereas a follow-up at 12 months may be useful. Thirdly, the study was conducted in a single center, and this could have limited the applicability of the results.

In conclusion, Hyalubrix 30 mg/2 mL and DIART 1.8%/2 mL are two safe and effective therapeutic options for the management of knee OA, offering benefits in pain control, functionality and emotional well-being. In the DIART 1.8%/2 mL formulation, the addition of succinate might work together with HA to boost pain relief, reduce inflammation, and enhance antioxidant effects.

## 4. Materials and Methods

### 4.1. Study Design

From October 2024 to May 2025, knee OA patients treated with linear HA formulations in the Pain Medicine Room of the Clinical Pharmacology and Pharmacovigilance Operative Unit at the “Renato Dulbecco” University Hospital in Catanzaro (Italy) were enrolled (1:1) in this prospective longitudinal single-center clinical study. Patients were evaluated at baseline (T0), during injections (one injection per week for three weeks, T1–T3), and three (T4) and six months later (T5). Before being admitted to the study, all enrolled patients signed an informed consent form at the start of the study and prior to first administration (T0). According to the 1964 Declaration of Helsinki and its subsequent changes, as well as Italian law, this study was carried out with complete regard for patient privacy (Ethics Committee authorization: 120/2018; ClinicalTrials.gov Identifier: NCT06814769).

### 4.2. Inclusion and Exclusion Criteria

Patients were enrolled according to the following eligibility criteria:

- Patients of both sexes over 18 years old;
- Patients with second- or third-degree OA, according to the Kellgren–Lawrence classification of a weight-bearing radiograph of the knee taken no more than one year ago;
- Patients with VAS (Visual Analogue Scale) > 5/10 refractory to systemic drug therapy;
- Patients who understood the study’s objectives and were able to provide their written informed consent to participate in the study and to use their data anonymously for scientific purposes.

Patients were excluded in the presence of the following conditions:

- Age < 18 years old;
- Presence of an active malignancy of any type or history of malignancy;
- Grade I arthritis according to the Kellgren–Lawrence criteria;
- Local or systemic infection;
- Uncooperative patients, those suffering from neurological disorders and who were therefore unable to follow the physician’s instructions or unable to provide informed consent for participation in the study, or those who did not provide written consent;
- Any cases not described in the inclusion criteria.

### 4.3. End Points

The primary efficacy endpoint was a statistically significant difference (*p* < 0.05) in VAS scores between T3 and T0 and between T4/T5 and T0.

Secondary endpoints included:

- a statistically significant difference (*p* < 0.05) in functional mobility and walking ability (six-minute walking test) between T3 and T0 and between T4/T5 and T0.
- a statistically significant difference (*p* < 0.05) in general health assessment (SF-36) between T3 and T0 and between T4/T5 and T0.
- a statistically significant difference (*p* < 0.05) in mood disorders (Zung SAS and Zung SDS) between T3 and T0 and between T4/T5 and T0.

### 4.4. Safety Endpoints

We recorded the emergence of adverse drug reactions (ADRs) associated with the intra-articular injection of HA throughout the study. In addition to ADRs that resulted in a subject’s withdrawal from the trial, we also recorded the frequency, predictability, length, severity, seriousness, course, and consequences of these ADRs.

### 4.5. Experimental Protocol

Clinical and laboratory data were collected directly by the physicians involved in the study after enrollment (T0) and during follow-ups (T1–T6), alongside the administration of Zung SAS, Zung SDS, and SF-36 questionnaires. In line with our studies, the specialized database assessed and documented any systemic or local side effects [15,26]. At the start of the study (T0), and thereafter every 10 days (up to a total of 3 injections), linear HA (molecular weight > 1500 kDa; DIART 1.8%/2 mL, Diaco Biofarmaceutici, Trieste, Italy or Hyalubrix 30 mg/2 mL, Fidia Farmaceutici 35,031 Abano Terme (PD), Italy) was given as a single injection. To rule out the occurrence of oedema, all recruited patients underwent knee echography prior to admission. Local corticosteroids were administered to edematous patients until the condition subsided. Lastly, a control group of OA patients receiving triamcinolone acetonide (40 mg/mL) and lidocaine (20 mg/mL; 2 mL) (three intra-articular injections every ten days) was used to assess the efficacy of the HA formulations.

### 4.6. Questionnaires

The 36-Item Short Form Health Survey (SF-36) was used to assess quality of life in relation to pathology and effectiveness of treatment. Physical functioning (PF), bodily pain (BP), role limitations due to physical health problems (RP), role limitations due to personal or emotional problems (RE), general mental health (MH), social functioning (SF), energy/fatigue or vitality (VIT), and general health perceptions (GH) are the eight concepts of health that are represented by the two component summary scores, the Physical Component Summary (PCS) and the Mental Component Summary (MCS), that are derived from the 36 questions. A higher score represents better health while a low score corresponds to a lower quality of life [55,56].

Zung’s Self-Rating Anxiety Scale (Zung SAS) is a 20-item questionnaire that measures the four typical psychological and physical aspects of anxiety. Responses are recorded using a 4-point rating system, where 1 represents “none or a little of the time” and 4 represents “most or all of the time.” Both good and bad events are included. The ultimate score falls between 20 and 80 points. Anxiety is classified as normal (score 0 to 44), moderate (score 45 to 59) and severe (score 60 to 80) [57].

Zung’s Self-Rating Depression Scale (Zung SDS) is a 20-item question scale that evaluates the four usual characteristics of depression. Items include psychological and physiological symptoms: 10 express negative experiences and 10 express positive experiences. Responses are given on a 4-point scale ranging from 1 (none or a little of the time) to 4 (most or all of the time). Total raw scores range from 20 to 80. Depression is classified as normal (score 20 to 49), mild (score 50 to 59), moderate (score 60 to 69) and severe (score 70 to 80) [57].

### 4.7. Statistical Analysis

Gaussian continuous variables were described by mean and standard deviation. Median and interquartile range were used in cases of skewness. Counts and percentages were used for categorical variables. The normal distribution of continuous variables was verified by the Shapiro–Wilk test. A *t*-test was used to compare normally distributed continuous variables between males and females, while the Mann–Whitney test was used in cases of skewness. A multivariate ANOVA (MANOVA) test was used to analyze the relationship between multiple dependent variables (comorbidity) and one independent variable (pain).

*p*-values < 0.05 were considered statistically significant. Statistical analysis was performed using SPSS 22.0 (International Business Machines Corporation, Armonk, NY, USA) and JASP (https://jasp-stats.org/), accessed on 1 April 2025 and 15 April 2025.

## Figures and Tables

**Table 1:** Demographic characteristics of the population. BMI, Body Mass Index; OA, osteoarthritis. Data are expressed as mean ± standard deviation for continuous variables and number (percentage) for categorical variables; * = *p* < 0.05.

	Hyalubrix 30 mg/2 mL (*n* = 35)	DIART 1.8%/2 mL (*n* = 35)
**Females**	20 (57.1)	18 (51.4)
**Males**	15 (42.9)	17 (48.6)
**BMI**	25.91 ± 2.73	25.86 ± 2.38
**Age**	58.71 ± 12.15	57.91 ± 7.97
**Knee OA (site)**		
Left	15 (42.9)	16 (45.7)
Right	20 (57.1)	15 (42.9)
Bilateral	0	4 (11.4)
**Kellgren–Lawrence grade**		
II	6 (17.1)	4 (11.4)
III	22 (62.9)	22 (62.9)
IV	7 (20.0)	9 (25.7)
**Comorbidities**		
Hypertension	9 (25.7)	20 (57.1) *
Dysthyroidism	9 (25.7)	9 (25.7)
Diabetes Type 2	0	7 (20.0) *
Osteoporosis	16 (45.7)	9 (25.7)
Fibromyalgia	3 (8.6)	0
Neurologic disease	2 (5.7)	1 (2.9)
Orthopedic disease	1 (2.9)	1 (2.9)
Anxiety/depression	4 (11.4)	2 (5.7)
Nephro-urologic disease	3 (8.6)	0
Gastroenterological disease	6 (17.1)	6 (17.1)
Dyslipidemia	3 (8.6)	10 (28.6) *
Rheumatological disease	0	3 (8.6)

**Table 2:** Treatments used before hyaluronic acid intra-articular treatment. NSAIDs, non-steroidal anti-inflammatory drugs. Student’s *t*-test was used for statistical evaluation; ** *p* < 0.01.

	Hyalubrix 30 mg/2 mL (*n* = 35)	DIART 1.8%/2 mL (*n* = 35)
**NSAIDs**	13 (37.1) **	6 (17.1)
**Opioids**	2 (5.7)	3 (8.6) **
**Corticosteroids**	5 (14.3) **	2 (5.7)
**Acetaminophen**	1 (2.9)	3 (8.6) **

**Table 3:** Demographic characteristics of OA patients enrolled in the control group and treated with triamcinolone acetonide (40 mg/mL) plus lidocaine (20 mg/mL; 2 mL). Data are expressed as mean ± standard deviation for continuous variables and number (percentage) for categorical variables. Data were evaluated using Student’s *t*-test and MANOVA test. ** *p* < 0.01.

	Man	Women
Number (%)	32 (64.0) **	18 (36.0)
Age	46.38 ± 12.27	47.59 ± 11.93
Body mass index (kg/m^2^)	26.40 ± 1.98	25.55 ± 1.79
Mean symptom duration (years)	4.59 ± 3.76 years	5.86 ± 4.12 years **
Osteoarthritis; Kellgren–Lawrence classification	n (%)	n (%)
Stage I	6 (18.8)	4 (22.2)
Stage II	25 (78.1)	13 (72.2)
Stage II/III	1 (3.1)	1 (5.6)
Stage III	0	0

**Table 4:** Clinical and functional scales at 1 week (T1), 2 weeks (T2), and 3 weeks (T3) vs. admission (T0) in patients treated with linear hyaluronic acid (test groups). Data are expressed as median (interquartile range). ** *p* < 0.01.

Score	T0	T1	PT1 vs. T0	T2	P T2 vs. T0	T3	P T3 vs. T0
Visual Analogue Scale							
Hyalubrix	7.69 ± 0.90	4.20 ± 0.63	0.000 **	1.57 ± 0.50	0.000 **	0.29 ± 0.46	0.000 **
DIART	7.89 ± 0.90	3.89 ± 0.76	0.000 **	1.77 ± 0.60	0.000 **	0.20 ± 0.47	0.000 **
KOOS							
Hyalubrix	47.34 ± 14.31	50.91 ± 15.17	0.000 **	56.97 ± 14.83	0.000 **	67.26 ± 10.32	0.000 **
DIART	49.49 ± 15.68	53.97 ± 16.82	0.000 **	61.00 ± 15.82	0.000 **	70.60 ± 12.18	0.000 **
Six-minute walking test							
Hyalubrix	336.20 ± 78.53	363.46 ± 81.93	0.000 **	396.10 ± 82.65	0.000 **	435.03 ± 77.83	0.000 **
DIART	348.77 ± 100.36	368.11 ± 101.43	0.000 **	401.77 ± 100.58	0.000 **	444.46 ± 95.60	0.000 **
Walking test visual analogue scale							
Hyalubrix	7.46 ± 1.38	7.26 ± 1.22	0.000 **	5.26 ± 1.27	0.000 **	1.94 ± 1.19	0.000 **
DIART	7.49± 1.46	7.31 ± 1.35	0.012 **	5.34 ± 1.33	0.000 **	1.74 ± 1.09	0.000 **
Timed up and go test (sec)							
Hyalubrix	9.37 ± 2.18	8.94 ± 2.13	0.000 **	8.34 ± 1.71	0.000 **	7.29 ± 1.15	0.000 **
DIART	9.17 ± 2.42	8.80 ± 2.55	0.026 **	8.34 ± 2.25	0.000 **	7.14 ± 1.59	0.000 **

**Table 5:** Clinical and functional scales at 3 months (T4) and 6 months (T5) vs. admission (T0) in patients treated with linear hyaluronic acid (test groups). Data are expressed as median (interquartile range). ** *p* < 0.01.

Score	T0	T4	PT4 vs. T0	T5	P T5 vs. T0
Visual analogue scale					
Hyalubrix 30 mg/2 mL	7.69 ± 0.90	0.80 ± 0.72	0.000 **	7.46 ± 0.66	0.073
DIART 1.8%/2 mL	7.89 ± 0.90	0.80 ± 1.08	0.000 **	6.83 ± 1.42	0.000 **
Knee Injury and Osteoarthritis Outcome score					
Hyalubrix 30 mg/2 mL	47.34 ± 14.31	78.71 ± 10.10	0.000 **	74.71 ± 10.38	0.000 **
DIART 1.8%/2 mL	49.49 ± 15.68	79.54 ± 13.83	0.000 **	75.63 ± 13.15	0.000 **
Six-minute walking test					
Hyalubrix	336.20 ± 78.53	439.26 ± 78.05	0.000 **	416.29 ± 77.86	0.000 **
DIART 1.8%/2 mL	348.77 ± 100.36	448.46 ± 95.99	0.000 **	435.74 ± 94.97	0.000 **
Walking test visual analogue scale					
Hyalubrix 30 mg/2 mL	7.46 ± 1.38	0.86 ± 1.19	0.000 **	3.57 ± 1.98	0.000 **
DIART 1.8%/2 mL	7.49 ± 1.46	0.60 ± 0.85	0.000 **	3.34 ± 1.43	0.000 **
Timed up and go test (sec)					
Hyalubrix 30 mg/2 mL	9.37 ± 2.18	7.06 ± 1.00	0.000 **	8.26 ± 1.34	0.004 **
DIART 1.8%/2 mL	9.17 ± 2.42	6.77 ± 1.21	0.000 **	8.26 ± 1.50	0.017 **

**Table 6:** Clinical and functional scales at 3 weeks (T3) vs. T0 in OA patients treated with triamcinolone acetonide (40 mg/mL) plus lidocaine (20 mg/mL; 2 mL) (control group). Data are expressed as median (interquartile range). KOOS: Knee Injury and Osteoarthritis Outcome score; VAS: Visual analogue scale.

Score	T0	T3	*p*
KOOS	46.35 ± 18.36	49.58 ± 15.21	0.340
VAS	6.78 ± 1.35	7.1 ± 2.18	0.379
Six-minute walking test (meters)	450 ± 126	461 ± 115	0.649
Walking test VAS	7.52 ± 1.25	6.98 ± 2.16	0.223
Timed up and go test (sec)	7.6 ± 2.3	5.51 ± 1.98	0.209

**Table 7:** Short Form Health Survey 36 and Zung’s scales recorded in enrolled OA patients at 3 weeks (T3) after admission (T0) in patients treated with linear hyaluronic acid (test groups). Data are expressed as mean ± standard deviation. ** *p* < 0.01.

SF-36						
	Hyalubrix 30 mg/2 mL		*p*	DIART 1.8%/2 mL		*p*
	T0	T3		T0	T3	
Physical functioning	38.38 ± 15.31	51.29 ± 7.31	0.000 **	42.43 ± 14.06	54.00 ± 8.72	0.000 **
Role limitations due to physical health	46.32 ± 23.14	72.86 ± 18.56	0.000 **	47.90 ± 22.99	73.57 ± 17.09	0.000 **
Role limitations due to emotional problems	40.94 ± 25.69	59.05 ± 25.69	0.000 **	41.89 ± 23.37	65.73 ± 22.13	0.000 **
Energy/fatigue	44.86 ± 13.58	55.14 ± 13.20	0.000 **	49.29 ± 10.86	59.57 ± 11.66	0.000 **
Emotional well-being	45.83 ± 10.15	53.14 ± 10.16	0.000 **	48.57 ± 11.36	59.54 ± 12.19	0.000 **
Social functioning	52.14 ± 13.38	61.43 ± 15.27	0.000 **	48.36 ± 16.46	58.93 ± 16.76	0.000 **
Pain	50.71 ± 10.28	59.21 ± 9.39	0.001 **	55.64 ± 12.75	68.80 ± 12.21	0.000 **
General health	50.57 ± 13.71	60.71 ± 13.73	0.000 **	46.86 ± 13.18	57.00 ± 13.46	0.000 **
Health change	44.29 ± 16.14	60.71 ± 17.45	0.000 **	45.86 ± 18.53	64.43 ± 15.09	0.000 **
Zung’s scales						
Depression	60.83 ± 6.61	52.60 ± 7.23	0.000 **	61.86 ± 11.33	53.97 ± 10.80	0.000 **
Anxiety	60.77 ± 7.71	52.20 ± 8.40	0.000 **	58.51 ± 10.15	51.83 ± 9.55	0.000 **

**Table 8:** Short Form Health Survey 36 and Zung’s scales recorded in enrolled OA patients at 3 months (T4) after admission (T0) in patients treated with linear hyaluronic acid (test groups). Data are expressed as mean ± standard deviation. ** *p* < 0.01.

SF-36						
	Hyalubrix 30 mg/2 mL		*p*	DIART 1.8%/2 mL		*p*
	T0	T4		T0	T4	
Physical functioning	38.38 ± 15.31	57.14 ± 7.10	0.000 **	42.43 ± 14.06	59.71 ± 8.22	0.000 **
Role limitations due to physical health	46.32 ± 23.14	68.57 ± 20.42	0.015 **	47.90 ± 22.99	70.00 ± 18.98	0.000 **
Role limitations due to emotional problems	40.94 ± 25.69	78.11 ±21.30	0.000 **	41.89 ± 23.37	80.97 ± 18.56	0.000 **
Energy/fatigue	44.86 ± 13.58	60.14 ± 13.20	0.000 **	49.29 ± 10.86	64.57 ± 11.66	0.000 **
Emotional well-being	45.83 ± 10.15	61.14 ± 10.16	0.000 **	48.57 ± 11.36	67.54 ± 12.19	0.000 **
Social functioning	52.14 ± 13.38	70.71 ± 12.84	0.000 **	48.36 ± 16.46	71.07 ± 17.09	0.000 **
Pain	50.71 ± 10.28	63.36± 9.66	0.002 **	55.64 ± 12.75	74.29 ± 12.12	0.000 **
General health	50.57 ± 13.71	70.71± 13.73	0.000 **	46.86 ± 13.18	67.00 ± 13.46	0.000 **
Health change	44.29 ± 16.14	78.57 ± 15.03	0.001 **	45.86 ± 18.53	83.57 ± 13.48	0.000 **
Zung’s scales						
Depression	60.83 ± 6.61	55.91 ±7.22	0.000 **	61.86 ± 11.33	56.60 ± 10.70	0.000 **
Anxiety	60.77 ± 7.71	55.400 ± 8.35	0.000 **	58.51 ± 10.15	56.37 ± 9.65	0.009 **

**Table 9:** Short Form Health Survey 36 and Zung’s scales recorded in enrolled OA patients at 6 months (T5) after admission (T0) in patients treated with linear hyaluronic acid (test groups). Data are expressed as mean ± standard deviation. ** *p* < 0.01.

SF-36						
	Hyalubrix 30 mg/2 mL		*p*	DIART 1.8%/2 mL		*p*
	T0	T5		T0	T5	
Physical functioning	38.38 ± 15.31	53.86 ± 9.24	0.000 **	42.43 ± 14.06	54,00 ± 9.46	0.000 **
Role limitations due to physical health	46.32 ± 23.14	57.86 ± 19.90	0.000 **	47.90 ± 22.99	55.71 ± 19.26	0.078
Role limitations due to emotional problems	40.94 ± 25.69	69.54 ± 21.96	0.000 **	41.89 ± 23.37	68.58 ± 24.19	0.000 **
Energy/fatigue	44.86 ± 13.58	50.29 ±14.85	0.001 **	49.29 ± 10.86	57.43 ± 11.91	0.000 **
Emotional well-being	45.83 ± 10.15	57.14 ± 10.16	0.000 **	48.57 ± 11.36	63.54 ± 12.19	0.000 **
Social functioning	52.14 ± 13.38	56.07 ± 12.27	0.086	48.36 ± 16.46	55.00 ± 14.28	0.005 **
Pain	50.71 ± 10.28	53.36 ± 9.66	0.002 **	55.64 ± 12.75	64.29 ± 12.12	0.000 **
General health	50.57 ± 13.71	55.71 ± 13.73	0.000 **	46.86 ± 13.18	52.00 ± 13.46	0.000 **
Health change	44.29 ± 16.14	28.571 ± 15.03	0.001 **	45.86 ± 18.53	33.57 ± 13.48	0.000 **
Zung’s scales						
Depression	60.83 ± 6.61	59.74 ± 6.64	0.000 **	61.86 ± 11.33	62.71 ± 14.09	0.626
Anxiety	60.77 ± 7.71	59.63 ± 8.00	0.000 **	58.51 ± 10.15	52.34 ± 15.27	0.002 **

**Table 10:** Short Form Health Survey 36 and Zung’s scales recorded 3 weeks (T3) after admission (T0), in OA patients treated with triamcinolone acetonide (40 mg/mL) plus lidocaine (20 mg/mL; 2 mL) (control group). Data are expressed as mean ± standard deviation.

SF-36			
	T0	T3	*p*
Physical functioning	51.35 ± 10.22	52.51 ± 10.16	0.570
Role limitations due to physical health	57.63 ± 12.65	56.95 ± 9.21	0.759
Role limitations due to emotional problems	50.26 ± 9.25	51.62 ± 8.81	0.453
Energy/fatigue	54.51 ± 8.43	56.15 ± 12.23	0.436
Emotional well-being	61.40 ± 8.56	62.26 ± 7.61	0.596
Social functioning	59.15 ± 8.85	60.41 ± 11.24	0.534
Pain	50.15 ± 10.27	49.12 ± 7.43	0.566
General health	47.56 ± 12.89	48.56 ± 10.12	0.667
Health change	52.10 ± 9.54	51.49 ± 7.55	0.354
Zung’s scales			
	T0	T3	*p*
Depression	63.51 ± 7.36	62.50 ± 7.91	0.523
Anxiety	55.23 ± 9.22	54.76 ± 9.14	0.798

## Data Availability

The original contributions presented in this study are included in the article. Further inquiries can be directed to the corresponding author.
